# Adverse Effects on Work and Daily Life Interference among Healthcare Workers after the First and Second ChAdOx1 and BNT162b2 COVID-19 Vaccine Doses

**DOI:** 10.3390/vaccines9080926

**Published:** 2021-08-19

**Authors:** Chulyong Park, Joon Sakong, Seongmin Jo, Minkeun Kim, Kiook Baek

**Affiliations:** 1 Department of Occupational and Environmental Medicine, Yeungnam University Hospital, Daegu 42415, Korea; ironyong@gmail.com (C.P.); jsakong@yu.ac.kr (J.S.); josm1129@ymc.yu.ac.kr (S.J.); 21420007@ynu.ac.kr (M.K.); 2 Department of Preventive Medicine and Public Health, College of Medicine, Yeungnam University, Daegu 42415, Korea; 3 Department of Occupational and Environmental Medicine, Korea University Medical Center Ansan Hospital, Ansan 15355, Korea

**Keywords:** COVID-19, side effect, vaccine

## Abstract

In this study, we assessed the adverse effects and the work and daily life interference associated with each dose of the ChAdOx1 and BNT162b2 COVID-19 vaccines. Questionnaires were distributed to workers after they received both doses; only those who worked the day after receiving the vaccine were included in the analysis. Overall, 368 ChAdOx1-vaccinated and 27 BNT162b2-vaccinated participants were included. Among the ChAdOx1-vaccinated participants, the incidence of adverse effects was significantly lower after the second dose than after the first dose. Among the BNT162b2-vaccinated participants, however, no differences in adverse effects or work and daily life interference were found between the doses. After the first and second dose, the numeric scale score (0–10) for interference with work was 3.9 ± 2.9 and 1.6 ± 1.9 for the ChAdOx1 and 3.2 ± 2.5 and 3.6 ± 3.0 for the BNT162b2 vaccine, respectively. A similar trend was observed for interference with daily life. Factors associated with work and daily life interference in the multivariate model were age, vaccine dose (first or second), and the interaction term of vaccine type and dose. These results could be used to inform the general population of the adverse effects associated with these vaccinations.

## 1. Introduction

As of 15 June 2021, over 15 million COVID-19 vaccination doses have been administered in the Republic of Korea [1], which represents approximately 30% of the population. With the increase in vaccination rates, many cases of adverse effects have been reported, including those that are common and less severe [2,3] and those that are more rare, but critical [4,5]. The reported rates of adverse effects have been inconsistent [6,7] because of differences in age, ethnicity, and underlying diseases. Therefore, research on these issues are ongoing.

A few previous studies conducted in the Republic of Korea have focused on the rate of local and systemic adverse effects [2,8]. However, the widely used system for reporting these rates is based on a binary answer (yes or no) for each adverse effect, making it difficult to know the degree of severity and their impact on work and daily life [9]. We therefore utilized a numeric scale (0–10) to determine the severity of the adverse effects and the extent to which they interfered with work and daily life. This numeric scale is widely used to assess the influence of a group of certain types of diseases on daily life [10,11,12] and can be understood intuitively.

For the general population, providing detailed information on adverse effects is essential for communicating the risks associated with these vaccinations [13]; however, the data currently available are insufficient. Detailed information regarding adverse effects, their severity, and their impact on individuals’ work and daily lives are required to educate the general population and encourage them to participate in vaccination. Therefore, we identified the adverse effects associated with the ChAdOx1 and BNT162b2 COVID-19 vaccinations and their effects on work performance and daily life.

## 2. Materials and Methods

### 2.1. Study Design and Participants

Hospital workers at a university hospital in Daegu, Republic of Korea, received their first dose of ChAdOx1 COVID-19 vaccine between 5 March 2021, and 26 March 2021, and the second dose 12 weeks later. The first and second doses of the BNT162b2 COVID-19 vaccine were administered in March, four weeks apart, to those who were working in the designated COVID-19 ward. The doses and methods of administration were determined according to the manufacturers’ instructions. An internet-based survey using NAVER Form (NAVER Corp., Bundang, Korea) was sent via mobile text messaging three times (from 2 June to 18 June) after the second vaccination dose was received. Since our main objective was to determine the association between the vaccination and interference with work and daily life, only subjects who worked the day after vaccination were included, and surveys with missing values among the variables of interest were excluded.

### 2.2. Survey

We collected information on sex, age group, vaccine type (i.e., BNT162b2 or ChAdOx1), previous illnesses, job class (medical staff or other), shift work, alcohol consumption, smoking status, regular exercise, and adverse effects after vaccination. Subjects were instructed to check ‘yes’ on the smoking and alcohol consumption questions if they had smoked or consumed alcohol at least once between March and May 2021 for the survey conducted after the first dose, and at least once between the first and second dose for the survey conducted after the second dose. For the exercise questionnaire, subjects were instructed to check ‘yes’ if they exercised for >150 min/week between March and May 2021 for the survey conducted of the first dose, and at least once between the first and second dose for the survey of the second dose. Furthermore, the intake of antipyretics, intravenous treatment with antipyretics or fluid, interference with work, interference with daily life, and medications after the first and second vaccination doses were determined using a structured questionnaire. A total of 10 adverse effects were included in the survey: fever, chills, localized pain, myalgia, headache, nausea, vomiting, urticaria, dyspnea, and chest pain. If the subject experienced symptoms other than those included in the questionnaire, they were instructed to write the symptoms under “other symptoms.” The subjective degree of severity for each adverse effect was collected using an 11-point (0–10) numeric scale, with 0 indicating no symptoms and 10 indicating the most severe symptoms. Information on work and daily life interference was also collected using an 11-point (0–10) numeric scale, with a score of 10 indicating no work or daily activities could be performed, and a score of 0 indicating no adverse effects. Information on work productivity was collected using an 11-point (0–10) numeric scale, with 10 indicating a usual level of work performance. The questionnaires were collected anonymously.

### 2.3. Statistical Analyses

Baseline characteristics, adverse effects, and response to adverse effects were described for each vaccine and vaccination dose (first and second). Adverse effects after the first and second doses were compared using a generalized linear mixed model. A logit link for categorical variables and a linear mixed model for continuous variables were used since repeated measures were considered for the participants. The adverse effects associated with the ChAdOx1 and BNT162b2 vaccines were compared for each dose (first and second) using the Fisher’s exact test for categorical variables and independent *t*-test for continuous variables.

The level of interference with daily life and work was determined, and the median score for each variable was classified into the “low” (0–4) or “high” (5–10) group. The *p*-value was calculated for continuous variables. A generalized linear mixed model was used to calculate the odds ratios (ORs) and *p*-values.

Each precipitating factor that could have affected daily life and work interference was analyzed using a generalized mixed model (logit link) in the univariate analysis, which considered repeated measures for the participants according to the first and second vaccination doses. ORs for each factor were calculated separately. A multivariate analysis with a generalized linear mixed model (logit link) was then performed, which included independent variables that may have been associated with the independent variables (*p* < 0.25), using a generalized mixed model (logit link). The Wald method was used to calculate the 95% confidence intervals (CIs) of the generalized linear mixed models. SPSS 20.00 (IBM, NY, USA) was used for the Fisher’s exact test and independent *t*-test analyses. R project version 4.1.0 (package “lme4”) was used for the generalized linear mixed model.

## 3. Results

### 3.1. General Characteristics of the Participants According to Vaccine Type and Dose

There were 1670 and 140 workers who received the ChAdOx1 and BNT162b2 COVID-19 vaccines, respectively. A total of 542 individuals responded to the questionnaire. Subjects who did not agree to participate in the study (*n* = 20), those who had missing values (*n* = 9), and those who did not work on the day after both doses (*n* = 118) were excluded. Since a primary objective of this study was to assess the association between the vaccine and interference with work, those who did not work on the day after receiving the vaccine for both doses were excluded from the analysis. Finally, a total of 395 workers were included, and 644 vaccination cases were observed (first and second doses separately). Therefore, subjects were included in the analysis only once, and their two doses were assessed separately. For the ChAdOx1 vaccine, a total of 299 cases were included after the first dose and 304 after the second dose, and for the BNT162b2 vaccine, 19 cases were included after the first dose and 22 after the second dose. The general characteristics of the participants who received the first and second vaccinations are shown in Table 1. Except for the proportions of those who performed shift work, there were no significant differences in the general characteristics between each vaccine dose and type. There were no previously confirmed cases of COVID-19 among the vaccinated participants in the center, and no critical adverse events, such as death, thrombosis, or myocarditis, were reported at the center.

### 3.2. Vaccine Adverse Effects and Impact on Daily Life and Work

Table 2 shows the characteristics of the adverse effects experienced by the participants and their responses according to vaccine dose and type. For the ChAdOx1 vaccine, all rates of symptoms, except urticaria, were higher for the first dose than for the second dose. There was also a significant difference in the proportion of subjects who used analgesics or received fluid and injection treatments. The differences in the numeric scale scores for interference with work and daily life and for work performance were also statistically significant. In contrast, among the BNT162b2-vaccinated participants, the rates of symptoms were similar between the first and second doses. There were also no significant differences between the first doses of the ChAdOx1 and BNT162b2 vaccines in terms of adverse effects or interference with daily life or work. The rates of fever, chills, and myalgia were significantly higher after the second dose of the BNT162b2 vaccine than after the second dose of the ChAdOx1 vaccine. The subjective symptoms reported by more than one participant were dizziness (*n* = 4), sore throat (*n* = 2), and numbness in the vaccinated arm (*n* = 2) in the ChAdOx1 vaccination group. Others reported nasal stuffiness, sleepiness, feeling dazed, voice changes, cold feet, and shortness of menstruation cycle.

The numeric scale scores for the effects of vaccination on daily life and work were also significantly higher in the BNT162b2 vaccination group than in the ChAdOx1 vaccination group. The numeric scale for work performance was also significantly lower after the second dose in the BNT162b2-vaccinated participants than in the ChAdOx1-vaccinated participants. Most respondents presented symptoms within 24 h of vaccination. The duration of adverse effects varied among individuals, with approximately 50% of the participants responding that they were affected for more than 24 h.

### 3.3. Associations between Adverse Effects after Vaccination and Work and Daily Life Inteference

Table 3 shows the adverse effects after the ChAdOx1 vaccination, which was the most common vaccination received by our subjects, according to the degree of interference with work and daily life.

For the ChAdOx1 vaccination, those who experienced high levels of interference with daily life (5–10 points) had high rates for all adverse effects except urticaria. Similarly, those who experienced high levels of interference with work (5–10 points) reported all side effects (except urticaria) more frequently, with the three most common adverse effects being local pain (83.2%), myalgia (78.3%), and fever (73.3%). In those who experienced high levels of interference with daily life, the three most common adverse effects were local pain (84.4%), myalgia (77.7%), and fever (70.9%). Myalgia, fever, chills, and local pain were the four most common adverse effects among both the high interference with work and high interference with daily life groups, among those who received the BNT162b2 vaccination. However, due to the relatively small sample size and small proportions of adverse effects among those who received the BNT162b2 vaccination, we did not calculate *p*-values. The results are presented in Supplementary Table S1.

### 3.4. Precipitating Factors Associated with Daily Life and Work Interference

Table 4 shows the results of the statistical analysis between precipitating factors and daily life and work interference. Vaccine type, dose (first or second), age, sex, shift work, job class (medical staff/other), alcohol consumption, smoking status, and regular exercise (>150 min/week) were selected as precipitating characteristics that could potentially be associated with interference in daily life and work. The second dose, age, shift work, and job class were associated with work interference in the univariate analysis. The second dose (OR = 0.09, *p* < 0.0001), age (OR = 0.22, *p* < 0.01 for 50–59 years, OR = 0.06, *p* < 0.01 for >60 years, compared with 20–29 years), and the interaction term “vaccine type (BNT162b2) * second dose” (OR = 20.29, *p* < 0.05) were significantly associated with work interference after vaccination in the multivariate analysis. The second dose and age >40 years were associated with lower work interference. Vaccine dose, age, shift work, job class, and sex were associated with daily life interference in the univariate analysis. In the multivariate analysis, age, vaccine type, vaccine dose (first or second), interaction term of vaccine type and dose, shift work, and job class were selected as independent variables. The results showed that age (OR = 0.25, *p* < 0.01 for 50–59 years, OR = 0.03, *p* < 0.01 for ≥ 60 years, compared with 20–29 years), second dose (OR = 0.11, *p* < 0.0001), and the interaction term “vaccine type (BNT162b2) * second dose” (OR = 20.70, *p* < 0.05) were significantly associated with work interference after vaccination. Age >40 years and second dose were associated with lower scores for interference with daily life. The interaction term of vaccine type and dose showed significantly higher ORs in both analyses, indicating that a different pattern was demonstrated in the first and second doses according to the vaccine type.

## 4. Discussion

In this study, a survey was conducted, which focused on the adverse effects and interference with daily life and work experienced by workers at our institution the day after receiving the first and second doses of the ChAdOx1 and BNT162b2 vaccinations. The adverse effects after vaccination for each vaccine type and dose, degree of interference with life or work, and associated factors were analyzed.

Different patterns of adverse effects, according to the vaccine type and dose, were observed in our study. After the first ChAdOx1 vaccination dose, an interference with work and daily life, indicated by an average score of 3.9/10 and 4.2/10 was noted, respectively, while after the second dose, the average interference scores were 1.7 and 1.6, respectively. Multiple regression analysis showed that work and daily life interference was low in the ChAdOx1 vaccination group after the second dose. In this study, fewer adverse effects were reported after the second ChAdOx1 vaccination dose, and its effects on daily life and work were minimal. Regarding the BNT162b2 vaccine, there were no significant differences between the first and second doses in terms of interference with work or daily life. The second BNT162b2 vaccination dose showed comparable or more adverse effects than the first dose. A study in Italy also reported that there were no significant differences in the adverse effect rates after the first and second doses of the BNT162b2 vaccine [14]. Different patterns between the first and second doses according to the type of vaccine were also significant for work and daily life interference in the multivariate analysis. This is shown by the statistically significant and positive interaction term of the type of vaccine (BNT162b2) and dose (second) (work interference, OR = 20.29; daily life interference, OR = 20.27), while the second dose alone had a negative effect size (OR = 0.09) [15]. Overall, this study found that adverse effects were mild after the second dose of the ChAdOx1 vaccine, and that different expression patterns were evident according to dose and vaccine.

Although determining the incidence of each side effect of the vaccine was not a main objective of our study, it is interesting to compare these results with those of previous studies from other countries. In this study, the incidences of fever, myalgia, and headache were 58.2, 60.2, and 45.2% after the first dose of the ChAdOx1 vaccine and 18.1, 20.7, and 22.4% after the second dose, respectively. The incidences of fever, myalgia, and headache were 31.6, 31.6, and 26.3% after the first dose of the BNT162b2 vaccine and 50.0, 45.5, and 36.4% after the second dose, respectively. Although it is difficult to directly compare our results to those of previous studies due to different study designs, the rate of adverse effects reported in the Republic of Korea was relatively high. Bae et al. [2] reported that 93.3% of ChAdOx1-vaccinated subjects and 80.1% of BNT162b2-vaccinated subjects experienced more than one adverse effect after the first dose. Among the common symptoms, fever occurred in 51.3%, headache in 69.5%, and muscle ache in 79.9% after ChAdOx1 vaccination. Studies from the same centers in the Republic of Korea reported that 89.1% experienced adverse effects after the second dose of the BNT162b2 vaccine. For frequent adverse effects, the incidence rate was 32.1% for fever, 69.1% for muscle ache, and 48.7% for headache [16]. Kim et al. [8] found that 90.9 and 52.5% of ChAdOx1- and BNT162b2-vaccinated subjects, respectively, experienced adverse effects after the first dose. Fever was present in 36.1%, headache in 47.4%, and myalgia in 60.5% after ChAdOx1 first dose. After the BNT162b2 vaccine, the adverse effect rates were relatively low, with fever reported in 5%, myalgia in 11.2%, and headache in 7.5%. In a study conducted in Nepal, after receiving the first dose of the ChAdOx1 vaccine, symptoms appeared in 91.6% of subjects, with fever reported in about 30%, headache in about 30%, and myalgia in about 25% (only presented using a bar graph) [17]. Abu-Hammad et al. [18] reported the adverse effect rates after the COVID-19 vaccinations in Jordan, and the incidences of fever, myalgia, and headache were 73.7, 21.3, and 27.7%, respectively, after the first dose of the ChAdOx1 vaccine, while the incidences after the second dose were much lower, at 1.7, 0.6, and 1.1%, respectively, which were comparable to the results of our study. In a study from Saudi Arabia, after the ChAdOx1 vaccine, 31.3% reported headaches and 27.5% reported myalgia and joint pain [19]. A study from India reported that 12.5% experienced fever, 11.2% experienced muscle aches, and 17.4% experienced headaches in the first three days of receiving the first dose of the ChAdOx1 vaccine [20]. In a German study, the incidence of headaches and feverishness and chills after the first dose of ChAdOx1 was approximately 50% each (presented using a bar graph) [21]. The incidence of adverse effects among US healthcare workers was 21.99, 45.70, and 45.48% for fever, myalgia, and headaches, respectively [22]. According to a British study on the adverse effects associated with vaccinations, after the first dose of ChAdOx1, the incidences of fever, myalgia, and headache were 8.2, 7.0, and 22.8%, respectively, which appear to be relatively low. For the first dose of the BNT162b2 vaccine, the incidences of fever, myalgia, and headache were 1.5, 2.3, and 7.8%, respectively. For the second dose of the BNT162b2 vaccine, a slightly higher proportion reported fevers, myalgia, and headaches (3.8, 5.0, 13.2%, respectively) [3]. The incidence of adverse effects in an Italian study was 7.5 and 17.9% for fever and 23.1 and 32.3% for myalgia after the first and second doses of the BNT162b2 vaccine, respectively [14]. The subjects of the cited studies were not specifically skewed or biased in terms of age or other characteristics. Differences in ethnicity, immune status, and living environment, however, did appear to have affected the adverse effects. Although the mechanism is completely different, race and ethnicity are known to affect vaccine immunity and adverse effects to other vaccines, such as HPV and the smallpox vaccine [23,24]. Additionally, these inconsistencies might be due to the expression of symptoms associated with different cultures or languages. Nevertheless, the public may be confused by the differences in these results, which depend on the research design and subjects studied. While receiving clear information about vaccines is associated more with vaccine acceptance than directly citing the results of research from other countries [25], it is necessary to organize the data from each community for risk communication. Thus, we suggest that each country create vaccination-related guidelines or principles based on their own evidence.

According to the results reported by the ChAdOx1 vaccine manufacturer, fewer side effects have been found in older age groups [6]. Moreover, in a previous study from the Republic of Korea, the rate of adverse effects was low in older adults [8]. The differences by age were also shown in antibody production rates and COVID-19 protection rates [26,27]. There also appears to be a difference in immune reactions according to age. In this study, the rate of adverse effects was found to be greater in the younger age group, which is in line with previous studies. However, the degree of or sensitivity to symptoms may differ according to age [28]. Therefore, the lower rate of expression of each adverse effect may not reflect the discomfort experienced by older adults. The influence that the vaccines had on work and daily life was also reported to be higher in the younger age group. An important finding of this study was that interference with daily life and work tends to decrease with age.

Our study also had some limitations. Information on the adverse effects experienced and their severity was requested after the second dose of vaccination was completed using a self-administered survey questionnaire. Although this method can reduce loss to follow-up, recall bias may have been significant. With recall bias, subjects usually overestimate symptoms within the first two weeks, and the response is relatively stable thereafter [29]. Only the second dose of the ChAdOx1 vaccine was administered within two weeks of receiving the questionnaire since the first vaccination was 12 weeks before the survey and the second vaccination dose of BNT162b2 was administered four weeks after the first dose. Adverse effects after the second dose of the ChAdOx1 vaccine showed an incidence of adverse effects and a degree of interference with daily life and work that was rather low compared to those after the first dose of the ChAdOx1 vaccine and both doses of BNT162b2. Although recall bias may have impacted the effect size, the direction of the results was not likely affected. Notoriety bias was another limitation of this study. We distributed the survey to almost all vaccine recipients; however, those who had few adverse effects may not have participated in the study. Likewise, the more serious the adverse effects experienced, the more the tendency may have been to complete the survey. A third limitation of this study was the relatively small study population. To compensate for this, a mixed effects model was used to increase the statistical power by assessing each vaccination dose separately for each participant. However, rare adverse effects, such as thromboembolism, could not be assessed due to the small sample size. There could have been other confounders, as the proportion of respondents was skewed toward women and medical staff. Multivariate modeling in the analysis of precipitating factors and work/daily life interference were performed to overcome this limitation. In the Republic of Korea, there are relatively few individuals who have been administered other vaccines, such as JNJ-78436735 and mRNA-1273; therefore, we could not analyze the adverse effects of vaccines other than ChAdOx1 and BNT162b2.

Currently, there is an excess of information on vaccinations and their side effects from various sources, much of which is incorrect and inaccurate. Accurate information specific to each country and region is scarce. Given that the dissemination of inaccurate information negatively affects vaccine acceptance [30], providing clear information based on local data is important and may increase vaccine acceptance. In this study, adverse effects and precipitating factors were identified and the associations between the vaccine and interference with daily life and work were evaluated.

## 5. Conclusions

In this study, adverse effects and interference with work and daily life after the ChAdOx1 and BNT162b2 COVID-19 vaccinations were analyzed. After the first and second doses of the vaccines, the mean numeric scale score for interference with work was 3.9 and 1.9 for the ChAdOx1 vaccine and 3.2 and 3.6 for the BNT162b2 vaccine, respectively. The degree of work interference caused by the first dose of the ChAdOx1 vaccine and both doses of the BNT162b2 vaccine was comparable, but it was lower than that after the second dose of the ChAdOx1 vaccine. Interference with daily life showed a similar trend. The factors associated with a lower rate of interference with work and daily life after vaccination were older age (50–59 years, ≥60 years) and second dose. Different patterns of interference with work and daily life were observed according to the dose (first or second) and the vaccine (ChAdOx1 or BNT162b2). The method used to report these results was intended to be easier for the public to understand intuitively, compared with studies that report the incidence of each side effect. Thus, these findings can be effective for risk communication and education regarding vaccinations.

## Figures and Tables

**Table 1:** General characteristics of the study participants by vaccine type and dose.

	ChAdOx1 (*n* = 368)			BNT162b2 (*n* = 27)				
	First Dose (a)	Second Dose (b)	*p*-Value (a vs. b)	First Dose (c)	Second Dose (d)	*p*-Value (c vs. d)	*p*-Value (a vs. c)	*p*-Value (b vs. d)
	(*n* = 299)	(*n* = 304)		(*n* = 19)	(*n* = 22)			
Age (years)								
20–29	96 (32.1%)	87 (28.6%)	Reference	11 (57.9%)	10 (45.5%)	Reference	0.13	0.09
30–39	56 (18.7%)	56 (18.4%)	0.68	4 (21.1%)	7 (31.8%)	0.39		
40–49	54 (18.1%)	66 (21.7%)	0.21	1 (5.3%)	3 (13.6%)	0.33		
50–59	81 (27.1%)	83 (27.3%)	0.57	3 (15.8%)	2 (9.1%)	0.76		
≥60	12 (4.0%)	12 (3.9%)	0.82	0 (0.0%)	0 (0.0%)	-		
Sex								
Male	80 (26.8%)	80 (26.3%)	0.90	2 (10.5%)	2 (9.1%)	0.87	0.17	0.07
Female	219 (73.2%)	224 (73.7%)		17 (89.5%)	20 (90.9%)			
Previous illness								
No	243 (81.3%)	244 (80.3%)	0.75	16 (84.2%)	21 (95.5%)	0.25	1.00	0.09
Yes	56 (18.7%)	60 (19.7%)		3 (15.8%)	1 (4.5%)			
Alcohol consumption								
No	132 (44.1%)	136 (44.7%)	0.88	9 (47.4%)	11 (50.0%)	0.87	0.78	0.63
Yes	167 (55.9%)	168 (55.3%)		10 (52.6%)	11 (50.0%)			
Smoking								
No	275 (92.0%)	281 (92.4%)	0.83	17 (89.5%)	21 (95.5%)	0.48	0.70	1.00
Yes	24 (8.0%)	23 (7.6%)		2 (10.5%)	1 (4.5%)			
Exercise (>150 min/week)								
No	183 (61.2%)	186 (61.2%)	1.00	12 (63.2%)	15 (68.2%)	0.74	0.87	0.51
Yes	116 (38.8%)	118 (38.8%)		7 (36.8%)	7 (31.8%)			
Job class			0.72					
Other	104 (34.8%)	110 (36.2%)		2 (10.5%)	4 (18.2%)	0.49	<0.05	0.09
Medical staff	195 (65.2%)	194 (63.8%)		17 (89.5%)	18 (81.8%)			
Shift work			0.22					
No	123 (41.1%)	140 (46.1%)		3 (15.8%)	4 (18.2%)	0.84	<0.05	<0.05
Yes	176 (58.9%)	164 (53.9%)		16 (84.2%)	18 (81.8%)			

Participants who worked the day after receiving each vaccination dose were included separately for each dose. Some participants were included only for the first or second dose. A generalized linear mixed model was used to calculate the *p*-values of the repeated measures. An independent *t*-test was used to calculate the *p*-values of continuous variables for independent participants. Fisher’s exact test was used to calculate the *p*-values of categorical variables with <5 expected values in at least one category. Otherwise, the chi-square test was used to calculate the *p*-values. Previous illnesses include cardiovascular disease, endocrine disease, allergic diseases, and anemia. Nurses and doctors were classified as “medical staff.” Paramedics, office workers, facility workers, and other supportive workers in the hospital were classified as “other”.

**Table 2:** Adverse effects after the first and second doses of the ChAdOx1 and BNT162b2 vaccinations.

	ChAdOx1 (*n* = 368)			BNT162b2 (*n* = 27)				
	First Dose (a)	Second Dose (b)	*p*-Value (a vs. b)	First Dose (c)	Second Dose (d)	*p*-Value (c vs. d)	*p*-Value (a vs. c)	*p*-Value (b vs. d)
	(*n* = 299)	(*n* = 304)		(*n* = 19)	(*n* = 22)			
Fever								
No	125 (41.8%)	249 (81.9%)	<0.0001	13 (68.4%)	11 (50.0%)	0.24	0.23	<0.001
Yes	174 (58.2%)	55 (18.1%)		6 (31.6%)	11 (50.0%)			
Chills								
No	137 (45.8%)	264 (86.8%)	<0.0001	12 (63.2%)	13 (59.1%)	0.79	0.14	<0.01
Yes	162 (54.2%)	40 (13.2%)		7 (36.8%)	9 (40.9%)			
Local pain								
No	94 (31.4%)	130 (42.8%)	<0.01	6 (31.6%)	7 (31.8%)	0.99	0.99	0.32
Yes	205 (68.6%)	174 (57.2%)		13 (68.4%)	15 (68.2%)			
Myalgia								
No	119 (39.8%)	241 (79.3%)	<0.0001	13 (68.4%)	12 (54.5%)	0.37	<0.05	<0.05
Yes	180 (60.2%)	63 (20.7%)		6 (31.6%)	10 (45.5%)			
Headache			<0.0001					
No	164 (54.8%)	236 (77.6%)		14 (73.7%)	14 (63.6%)	0.49	0.11	0.13
Yes	135 (45.2%)	68 (22.4%)		5 (26.3%)	8 (36.4%)			
Nausea			<0.0001					
No	247 (82.6%)	292 (96.1%)		18 (94.7%)	20 (90.9%)	0.64	0.22	0.24
Yes	52 (17.4%)	12 (3.9%)		1 (5.3%)	2 (9.1%)			
Vomiting			<0.01					
No	284 (95.0%)	301 (99.0%)		18 (94.7%)	22 (100.0%)	1.00	1.00	1.00
Yes	15 (5.0%)	3 (1.0%)		1 (5.3%)	0 (0.0%)			
Urticaria			0.41					
No	295 (98.7%)	302 (99.3%)		19 (100.0%)	22 (100.0%)	-	1.00	1.00
Yes	4 (1.3%)	2 (0.7%)		0 (0.0%)	0 (0.0%)			
Dyspnea			<0.05					
No	289 (96.7%)	303 (99.7%)		18 (94.7%)	22 (100.0%)	1.00	0.50	1.00
Yes	10 (3.3%)	1 (0.3%)		1 (5.3%)	0 (0.0%)			
Chest pain			<0.05					
No	288 (96.3%)	301 (99.0%)		19 (100.0%)	22 (100.0%)	-	1.00	1.00
Yes	11 (3.7%)	3 (1.0%)		0 (0.0%)	0 (0.0%)			
Intake of PO antipyretics								
No	106 (35.5%)	158 (52.0%)	0.00	13 (68.4%)	9 (40.9%)	0.08	<0.01	0.38
Yes	193 (64.5%)	146 (48.0%)		6 (31.6%)	13 (59.1%)			
IV injection or fluid treatment			0.00					
No	271 (90.6%)	301 (99.0%)		18 (94.7%)	21 (95.5%)	0.92	1.00	0.25
Yes	28 (9.4%)	3 (1.0%)		1 (5.3%)	1 (4.5%)			
Latency period for overall adverse effects (h)								
Non-respondents	34 (11.4%)	71 (23.4%)		4 (21.1%)	4 (18.2%)		0.60	0.72
<6	37 (12.4%)	98 (32.2%)		6 (31.6%)	8 (36.4%)			
6–11	151 (50.5%)	88 (28.9%)		7 (36.8%)	7 (31.8%)			
12–23	61 (20.4%)	28 (9.2%)		2 (10.5%)	3 (13.6%)			
≥24	16 (5.4%)	19 (6.2%)		0 (0.0%)	0 (0.0%)			
Duration of overall adverse effects (h)							0.24	0.15
Non-respondents	32 (10.7%)	74 (24.3%)		5 (26.3%)	4 (18.2%)			
<6	34 (11.4%)	70 (23.0%)		1 (5.3%)	1 (4.5%)			
6–11	44 (14.7%)	26 (8.6%)		1 (5.3%)	2 (9.1%)			
12–23	44 (14.7%)	37 (12.2%)		3 (15.8%)	3 (13.6%)			
≥24	145 (48.5%)	97 (31.9%)		9 (47.4%)	12 (54.5%)			
Interference with work	3.9 ± 2.9	1.6 ± 1.9	<0.0001	3.2 ± 2.5	3.6 ± 3.0	0.57	0.30	<0.0001
Interference with daily life	4.2 ± 3.1	1.7 ± 2.1	<0.0001	3.5 ± 2.8	3.7 ± 3.1	0.78	0.34	<0.0001
Work performance	5.9 ± 2.8	7.0 ± 3.2	<0.0001	5.4 ± 2.9	4.8 ± 2.9	0.53	0.45	<0.01

A generalized linear mixed model was used to calculate the *p*-values of the repeated measures. An independent *t*-test was used to calculate the *p*-values for continuous variables. The Fisher’s exact test was used to calculate the *p*-values of categorical variables with <5 expected values in at least one category. Otherwise, the chi-square test was used to calculate the *p*-values. Abbreviations: PO, per os (oral administration); IV, intravenous.

**Table 3:** Association between severity of interference with work and daily life and presence of each adverse effect after ChAdOx1 vaccination.

		Interference with Work			Interference with Daily Life		
	Total	Low (0–4)	High (5–10)		Low (0–4)	High (5–10)	
	(*n* = 603)	(*n* = 442)	(*n* = 161)	*p*-value	(*n* = 449)	(*n* = 195)	*p*-value
Fever	229 (38.0%)	111 (25.1%)	118 (73.3%)	<0.0001	102 (24.1%)	127 (70.9%)	<0.0001
Chills	202 (33.5%)	90 (20.4%)	112 (69.6%)	<0.0001	82 (19.3%)	120 (67.0%)	<0.0001
Local pain	379 (62.9%)	245 (55.4%)	134 (83.2%)	<0.0001	228 (53.8%)	151 (84.4%)	<0.0001
Myalgia	243 (40.3%)	117 (26.5%)	126 (78.3%)	<0.0001	104 (24.5%)	139 (77.7%)	<0.0001
Headache	203 (33.7%)	102 (23.1%)	101(62.7%)	<0.0001	91 (21.5%)	112 (62.6%)	<0.0001
Nausea	64 (10.6%)	25 (5.7%)	39 (24.2%)	<0.0001	21 (5.0%)	43 (24.0%)	<0.0001
Vomiting	18 (3.0%)	2 (0.5%)	16 (9.9%)	<0.0001	3 (0.7%)	15 (8.4%)	<0.0001
Urticaria	6 (1.0%)	5 (1.1%)	1 (0.6%)	0.67	5 (1.2%)	1 (0.6%)	0.54
Dyspnea	11 (1.8%)	2 (0.5%)	9 (5.6%)	<0.0001	1 (0.2%)	10 (5.6%)	<0.0001
Chest pain	14 (2.3%)	3 (0.7%)	11 (6.8%)	<0.0001	3 (0.7%)	11 (6.1%)	<0.0001
Degree of total adverse effects	3.5 ± 2.8	2.3 ± 2.1	6.6 ± 1.7	<0.0001	2.2 ± 2.1	6.3 ± 1.9	<0.0001

*p*-values were calculated using a generalized linear mixed model, with the dependency of the repeated measures of the participants considered as a random effect. The degree of total adverse effects was assessed using a 0–10 numeric scale. Abbreviations: CI, confidence interval.

**Table 4:** Univariate and multivariate analyses of the precipitating factors associated with interference with work and daily life after vaccination.

		Interference with Work				Interference with Daily Life			
		Univariate Analysis		Multivariate Analysis		Univariate Analysis		Multivariate Analysis	
		OR (95% CI)	*p*-Value	OR (95% CI)	*p*-Value	OR (95% CI)	*p*-Value	OR (95% CI)	*p*-Value
Vaccine									
	ChAdOx1	Reference		Reference		Reference		Reference	
	BNT162b2	1.57 (0.69–3.60)	0.28	0.26 (0.06–1.16)	0.08	1.49 (0.68–3.25)	0.32	0.25 (0.06–1.09)	0.07
Dose									
	First	Reference		Reference		Reference		Reference	
	Second	0.09 (0.04–0.17)	<0.0001	0.06 (0.03–0.13)	<0.0001	0.11 (0.06–0.20)	<0.0001	0.08 (0.04–0.16)	<0.0001
Vaccine (BNT162b2) * dose (second)		-		20.29 (3.13–130.32)	<0.05	-		20.70 (3.32–129.02)	<0.05
Age (years)									
	20–29	Reference		Reference		Reference		Reference	
	30–39	0.85 (0.50–1.46)	0.57	1.01 (0.44–2.34)	0.98	0.77 (0.45–1.31)	0.33	0.76 (0.34–1.68)	0.50
	40–49	0.38 (0.21–0.70)	<0.05	0.38 (0.15–0.98)	0.45	0.46 (0.26–0.81)	<0.01	0.43 (0.18–1.02)	0.06
	50–59	0.25 (0.14–0.46)	<0.0001	0.22 (0.08–0.60)	<0.01	0.32 (0.19–0.57)	<0.0001	0.25 (0.10–0.62)	<0.01
	≥60	0.06 (0.01–0.47)	<0.01	0.03 (0.00–0.43)	<0.01	0.05 (0.01–0.43)	<0.01	0.03 (0.00–0.38)	<0.01
Sex									
	Male	Reference		Reference		Reference		Reference	
	Female	1.63 (0.97–2.72)	0.06	0.84 (0.37–1.93)	0.69	1.72 (1.05–2.77)	<0.05	1.15 (0.53–2.48)	0.72
Shift work									
	No	Reference		Reference		Reference		Reference	
	Yes	2.34 (1.48–3.74)	<0.001	1.79 (0.88–3.60)	0.11	1.57 (1.03–2.39)	<0.05	0.94 (0.50–1.79)	0.86
Job class									
	Other	Reference		Reference		Reference		Reference	
	Medical staff	2.39 (1.46–3.94)	<0.001	1.75 (0.79–3.90)	0.11	2.10 (1.32–3.29)	<0.01	1.54 (0.74–3.16)	0.25
Alcohol consumption									
	No	Reference		-		Reference		-	
	Yes	0.80 (0.52–1.23)	0.31	-		1.02 (0.68–1.54)	0.93	-	
Current smoker									
	No	Reference		-		Reference		-	
	Yes	1.17 (0.53–2.61)	0.69	-		1.09 (1.32–3.29)	0.81	-	
Exercise (>150 min/week)									
	No	Reference		-		Reference		-	
	Yes	0.74 (0.47–1.16)	0.19	-		1.00 (0.66–1.52)	0.99	-	

*p*-values and ORs were calculated using a generalized linear mixed model (logit link). For the dependent variable, “low” and “high” interference was defined as 0–4 and 5–10 for each questionnaire, respectively, divided by the median value 4. The OR was calculated as the odds of “high” interference over “low” interference. Abbreviations: OR, odds ratio; CI, confidence interval.

## Data Availability

The data presented in this study are available upon request from the corresponding author. The data are not publicly available due to restricted consent.

## Supplementary Materials

The following are available online at www.mdpi.com/article/10.3390/vaccines9080926/s1, Table S1: The association between severity of work and daily life interference and expression of each adverse effect after BNT162b2 vaccination.
