# Supplementary material for: Adverse Effects on Work and Daily Life Interference among Healthcare Workers after the First and Second ChAdOx1 and BNT162b2 COVID-19 Vaccine Doses
Source: Vaccines (Basel). 2021 Aug 19;9(8):926. doi: 10.3390/vaccines9080926 (PMC8402749; doi:10.3390/vaccines9080926)
Supplement: Supplementary file 1 [file vaccines-09-00926-s001.zip › vaccines-1303774-supplementary.pdf]

**Table S1.** The association between severity of work and daily life interference and expression of each adverse effect after BNT162b2 vaccination.

| | | Interference in work | | Interference in daily life | |
|---|---|---|---|---|---|
| | **Total** | **Low (0–4)** | **High (5–10)** | **Low (0–4)** | **High (5–10)** |
| | (N=41) | (N=26) | (N=15) | (N=26) | (N=15) |
| Fever | 17 (41.5%) | 4 (15.4%) | 13 (86.7%) | 2 (7.7%) | 15 (100.0%) |
| Chills | 16 (39.0%) | 4 (15.4%) | 12 (80.0%) | 4 (15.4%) | 12 (80.0%) |
| Local pain | 28(68.3%) | 16 (61.5%) | 12 (80.0%) | 15 (57.7%) | 13 (86.7%) |
| Myalgia | 16 (39.0%) | 3 (11.5%) | 13 (86.7%) | 3 (11.5%) | 13 (86.7%) |
| Headache | 13 (31.7%) | 3 (11.5%) | 10 (66.7%) | 3 (11.5%) | 10 (66.7%) |
| Nausea | 3 (7.3%) | 0 (0.0%) | 3 (20.0%) | 0 (0.0%) | 3 (20.0%) |
| Vomiting | 1 (2.4%) | 0 (0.0%) | 1 (6.7%) | 0 (0.0%) | 1 (6.7%) |
| Urticaria | 0 (0.0%) | 0 (0.0%) | 0 (0.0%) | 0 (0.0%) | 0 (0.0%) |
| Dyspnea | 1 (2.4%) | 0 (0.0%) | 1 (6.7%) | 0 (0.0%) | 1 (6.7%) |
| Chest pain | 0 (0.0%) | 0 (0.0%) | 0 (0.0%) | 0 (0.0%) | 0 (0.0%) |
| Degree of total adverse effects | 3.7 ± 2.6 | 2.3 ± 2.0 | 6.2 ± 1.6 | 2.2 ± 1.9 | 6.1 ± 1.7 |

The degree of total adverse effects was assessed using a 0–10 numeric scale.
Abbreviations: OR, odds ratio; CI, confidence interval
